# Resolving a century-old year mystery – the identity and provenance of the semiterrestrial crab, Parathelphusa (Liothelphusa) nobilii Colosi, 1920 (Decapoda, Brachyura, Gecarcinucidae) from Sarawak and a replacement name for *Parathelphusa nobilii* Ng, 2014

**DOI:** 10.3897/zookeys.1268.179174

**Published:** 2026-02-04

**Authors:** Peter K. L. Ng, Jongkar Grinang

**Affiliations:** 1 Lee Kong Chian Natural History Museum, Faculty of Science, National University of Singapore, 2 Conservatory Drive, Singapore 117377, Singapore Faculty of Science, National University of Singapore Singapore Singapore; 2 Institute of Biodiversity and Environmental Conservation, Universiti Malaysia Sarawak, 94300 Kota Samarahan, Sarawak, Malaysia Institute of Biodiversity and Environmental Conservation, Universiti Malaysia Sarawak Kota Samarahan Malaysia

**Keywords:** Borneo, distribution, freshwater crab, limestone karst, Malaysia, primary homonym, replacement name, taxonomy

## Abstract

The uncertain taxonomic identity and provenance of the gecarcinucid karst crab Parathelphusa (Liothelphusa) nobilii Colosi, 1920 is resolved through the examination of historical type material and newly collected specimens. *Stygothelphusa nobilii* (Colosi, 1920) is confirmed as a distinct species closely related to *S. bidiensis* Lanchester, 1900, but it can be easily distinguished by its more quadrate carapace, the anterolateral margin being armed with a low epibranchial tooth, proportionally shorter ambulatory legs, and diagnostic gonopod morphology. The originally stated type locality, “Mt Saribau” is considered erroneous. Evidence from the collector’s records and recent collections indicate that the species was very likely obtained from the limestone karsts around Gua Chupak in southwestern Sarawak. In addition, *Parathelphusa nobilii* Ng, 2014 from Sambas in Indonesian Kalimantan is identified a junior primary homonym of Parathelphusa (Liothelphusa) nobilii Colosi, 1920. A replacement name, *P. daisyae***nom. nov**., is therefore proposed.

## Introduction

The poorly known freshwater gecarcinucid crab Parathelphusa (Liothelphusa) nobilii Colosi, 1920 was originally described from three specimens reportedly collected by Robert Shelford at 2,500 feet above sea level on Mount Saribau, in Sarawak. No additional locality or ecological information was recorded, and the specimens bore no collection date. These specimens were first sent to Giuseppe Nobili at the Turin Museum, where they were initially identified as “Potamon (Geothelphusa) kenepai De Man, 1899” (Nobili 1903: 15).

The species was not treated until Bott’s (1970) revision of Asian freshwater crabs, where he synonymised it under *Thelphusula hendersoniana* (De Man, 1899) with only brief comments and without illustrations, although he apparently examined the specimens. Later, Ng (1991: 3), when establishing a new genus, *Arachnothelphusa* for Potamon (Potamon) melanippe De Man, 1899, suggested that Parathelphusa (Liothelphusa) nobilii might also belong to that genus and included it in his key (Ng 1991: 4). In 1998, a student of the first author, Oliver Chia, examined Colosi’s types at the Turin Museum and photographed the external morphology of the three specimens, although the gonopods were not imaged. Based on these photographs, Ng and Alvarez (2000: 337) noted that “a re-examination of the types indicates that it is a valid species of *Stygothelphusa* (unpublished data)”. *Stygothelphusa* had been established by Ng (1989a) for the cave-dwelling Potamon (Thelphusa) bidiense Lanchester, 1900 from Bau, Sarawak. *Stygothelphusa nobilii* was not discussed further until Ng (2013) described a third species of the genus from Serian, Sarawak, and figured the types of *S. nobilii* using the earlier photographs.

The male gonopods of Parathelphusa (Liothelphusa) nobilii have never been illustrated, and external morphological characters in freshwater crabs are often insufficient for determining generic, and sometimes even familial, placement. To confirm its generic identity and its validity as a species, the diagnostic male first and second gonopods must be examined directly.

The authors have been actively surveying karst landscapes and associated caves in Sarawak for more than two decades and have reported many species from these habitats (see Grinang and Ng 2015a, 2021; Ng 1989a, b, 2005, 2021; Ng and Earl of Cranbrook 2014; Ng and Grinang 2004, 2014). During the last decade, our work in the Gua Chupak karst region yielded specimens of a *Stygothelphusa* that superficially resemble the type of *S. nobilii* illustrated in Ng (2013), particularly in carapace shape and proportions of the ambulatory legs. However, without photographs of the epistome, mouthparts, male thoracic sternum, pleon, and especially the gonopods, we could not confirm whether the Gua Chupak material was conspecific with *S. nobilii*. This uncertainty was compounded by the questionable type locality, “Mount Saribau”, a site we were unable to locate on any modern map. Attempts to borrow and examine the types in Turin were unsuccessful for more than a decade because the specimens had been transferred to a basement storage area inaccessible due to health and safety restrictions, combined with limited staff and resources at the museum (see Grinang and Ng 2015b: 567).

After a hiatus of 28 years, the first author was finally able to examine the type specimens of Parathelphusa (Liothelphusa) nobilii during a visit to the Turin Museum in September 2025. With the crucial assistance of museum staff, including a newly appointed invertebrate curator who located and retrieved the types, we were able to study their morphology in detail. Comparisons with material recently collected from Gua Chupak show that the two are conspecific, with nearly identical gonopods. The present paper diagnoses this poorly known species based on both the type material and fresh specimens and provides detailed figures to facilitate its identification. We also discuss the probable true locality of “Mount Saribau” which we propose corresponds to the Gua Chupak-Mount Sibow region at the headwater of the Samarahan River, an area known to have been explored by Robert Shelford.

This study further establishes that Parathelphusa (Liothelphusa) nobilii is the senior primary homonym of *Parathelphusa nobilii* Ng, 2014 from Sambas in Indonesia Kalimantan. A replacement name, *P. daisyae* nom. nov., is therefore proposed for *Parathelphusa nobilii*.

## Materials and methods

Specimens examined are deposited in the zoological collections of the Institute of Biodiversity and Environmental Conservation, Universiti Malaysia Sarawak, Malaysia (**UNIMAS**); Museo Regionale di Scienze Naturali, Torino, Italy (**MRSN**) (previously Museum of Zoology in the University of Turin); and the Zoological Reference Collection of the Lee Kong Chian Natural History Museum, National University of Singapore (**ZRC**) (previously Raffles Museum of Biodiversity Research).

Comparative material examined includes *S. bidiensis* and *S. cranbrooki* as listed in Ng (1989 2013), and *S. antu* from Ng and Grinang (2014). Additional material of *Stygothelphusa cranbrooki* Ng, 2013 examined is as follows: MALAYSIA, Sarawak – limestone cave system, Gua Sireh, Kampung Bantang, Serian Division; coll. J. Grinang, 30 April 2016: 3 ♂ (14.1 × 12.4 mm, 13.4 × 12.2 mm, 13.6 × 12.3 mm), 4 ♀ (18.7 × 16.5 mm, 15.2 × 13.8 mm, 14.9 × 13.8 mm, 16.7 × 14.4 mm), 1 juvenile (8.4 × 7.0 mm) (UNIMAS.C.00066); 2 ♂ (19.1 × 16.8 mm, 13.5 × 12.8 mm) (ZRC 2021.0522).

The terminology used follows Ng (1988) and Davie et al. (2015). Measurements provided in millimetres are of the carapace width and length, respectively. The following abbreviations are used: **asl** = above sea level; **coll**. = collected by; **G1** = male first gonopod; **G2** = male second gonopod. The local Malay words, Gunung and Gua, are used for mountain and cave, respectively. The proportional ratio used in the key refers to the length of the fourth ambulatory merus relative to carapace length, with the fourth ambulatory merus length defined as the maximum length measured along the extensor margin.

## Taxonomy

### Superfamily Gecarcinucoidea Rathbun, 1904

**Family Gecarcinucidae Rathbun, 1904**

#### Genus Stygothelphusa

Taxon classificationAnimaliaDecapodaGecarcinucidae

Ng, 1989

1809B730-95BC-5970-9B1F-34E0990D453A

##### Type species.

Potamon (Thelphusa) bidiense Lanchester, 1900, by original designation and monotypy; gender feminine. The type locality is Bidi Cave, Bau District, Sarawak, Borneo.

##### Remarks.

Four species of *Stygothelphusa* are known: *S. antu* Ng & Grinang, 2014, *S. bidiensis* (Lanchester, 1900), *S. cranbrooki* Ng, 2013, and *S. nobilii*. With the examination of *S. nobilii*, a proper key to the members of the genus can now be provided (see below). The number of species of *Stygothelphusa*, however, will probably increase as more cave systems in western Sarawak are explored.

In recent papers, the structure of the male thoracic sternum has been shown to be different between allied gecarcunicid genera (Ng 2025; Ng and Grinang in press; Ng and Guinot in press), with the extent of the median longitudinal groove, presence of sternal bridges and structure of the median plate being useful. In male *Stygothelphusa*, sternite 5 is entire and medially fused; the tubercle of the press-button of the pleonal locking mechanism is on the proximal third of sternite 5; the median longitudinal groove extends over sternites 6–8, and is interrupted by transverse bridges between sternites 5 and 6, and sternites 6 and 7 (Fig. 6A, B).

#### Stygothelphusa nobilii

Taxon classificationAnimaliaDecapodaGecarcinucidae

(Colosi, 1920)

BFACF0DA-C6B0-5B93-BB7E-13EF9FACD3CB

Figs 1, 2, 3, 4, 5, 6, 7, 8, 9, 10, 11, 13A, E

Parathelphusa (Liothelphusa) nobilii Colosi, 1920: 26.
Thelphusula hendersoniana — Bott 1970: 59 (not Potamon (Geothelphusa) hendersonianum De Man, 1899).
Arachnothelphusa nobilii — Ng 1991: 1, 2.
Stygothelphusa nobilii — Ng 2004: 327; Ng and Yeo 2007: 18; Ng et al. 2008: 72; Cumberlidge et al. 2009: appendix 1; Ng 2013: 92–93, fig. 2.

##### Type material examined.

***Lectotype*** • ♂ (14.5 × 12.2 mm) (MRSN Cr 1308a, ex MZUT Cr 1570) [designated by Ng 2013], Mt. Saribau, Sarawak, 2500 feet, coll. R. Shelford, ca. 1902. ***Paralectotypes*** • 1 ♂ (17.1 × 14.7 mm), 1 ♀ (20.1 × 17.0 mm) (MRSN Cr 1308b, ex MZUT Cr 1570), same data as lectotype.

##### Other material examined.

Malaysia • 1 ♀ (16.2 × 14.5 mm) (ZRC 2017.1276), Gua Chupak, Tapah, Sarawak, Borneo, coll. PKL Ng & J. Grinang, June 2016 • 1 ♂ (17.6 × 15.5 mm), 1 ♀ (12.3 × 11.0 mm) (ZRC 2021.0661), 2 ♀ (12.4 × 11.4 mm, 10.4 × 8.9 mm) (UNIMAS.C.00064), Gua Chupak, Kampung Skuduk, Tapah, Siburan, Sarawak, coll. J. Grinang & C. K. Jongkar, 9 April 2016 • 1 young ♀ (7.8 × 7.0 mm) (ZRC 2021.0660), 2 juveniles (UNIMAS.C.00065), Gua Chupak, Kampung Skuduk, Tapah, Siburan, Sarawak, coll. J. Grinang & C. K. Jongkar, 12 December 2015.

##### Diagnosis.

**Figure 1.**
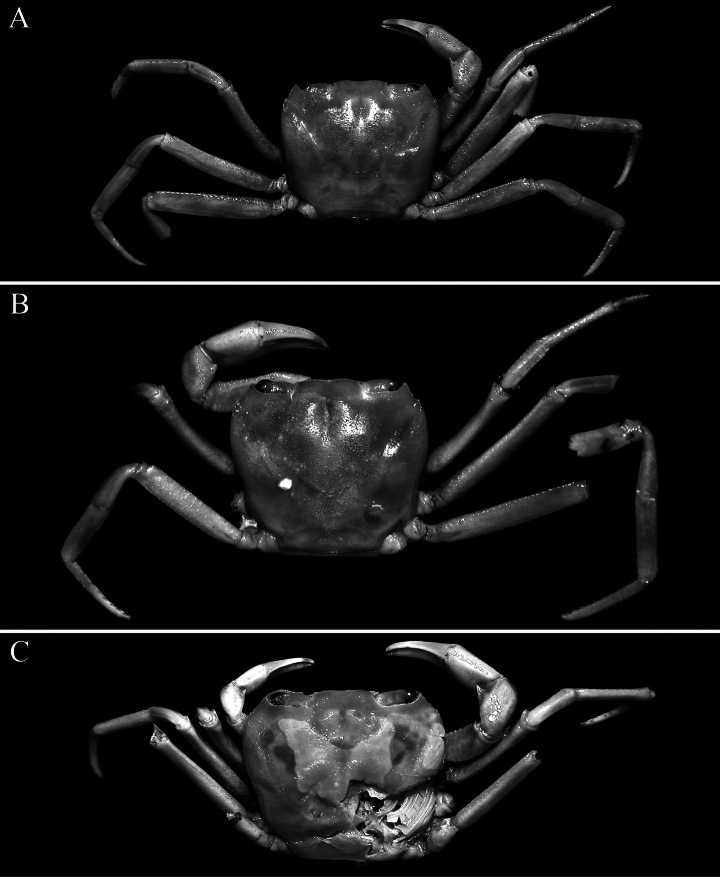
*Stygothelphusa nobilii* (Colosi, 1920), overall dorsal habitus. **A**. Lectotype ♂ (15.0 × 13.0 mm) (MRSN Cr 1308a); **B**. Paralectotype ♂ (17.0 × 14.7 mm) (MRSN Cr 1308b); **C**. Paralectotype ♀ (21.0 × 18.0 mm) (MRSN Cr 1308b). All specimens from Saribau, Sarawak.

**Figure 2.**
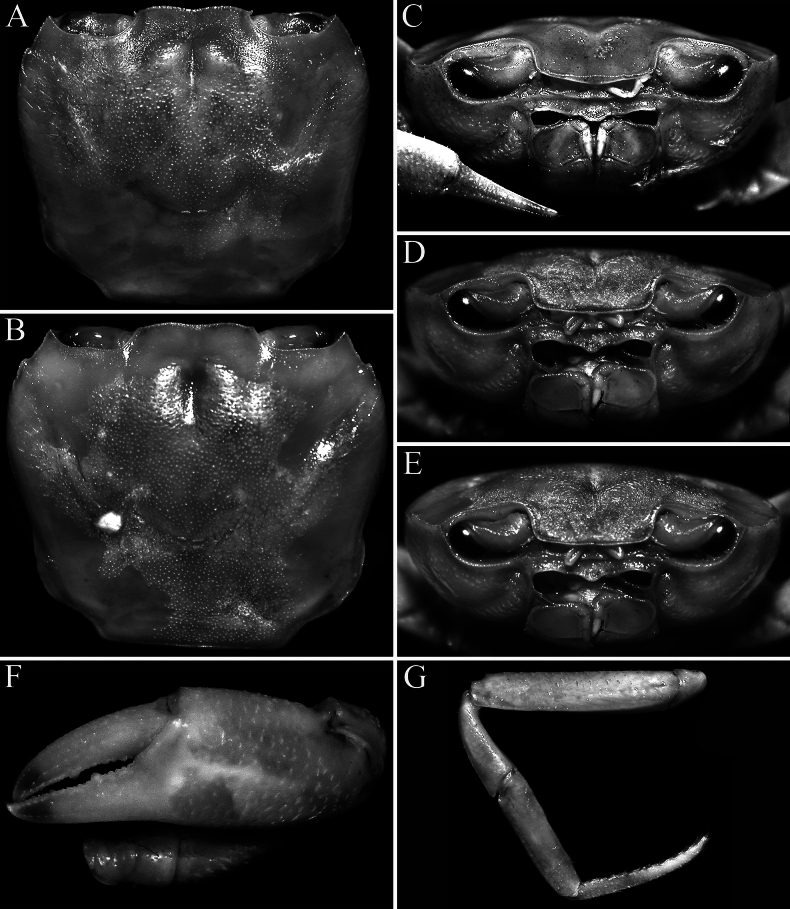
*Stygothelphusa nobilii* (Colosi, 1920). **A, C**. Lectotype ♂ (15.0 × 13.0 mm) (MRSN Cr 1308a); **B, D–G**. Paralectotype ♂ (17.0 × 14.7 mm) (MRSN Cr 1308b). Both specimens from Saribau, Sarawak. **A, B**. Dorsal view of carapace; **C–E**. Frontal view of cephalothorax; **F**. Outer view of left chela; **G**. Left P5.

**Figure 3.**
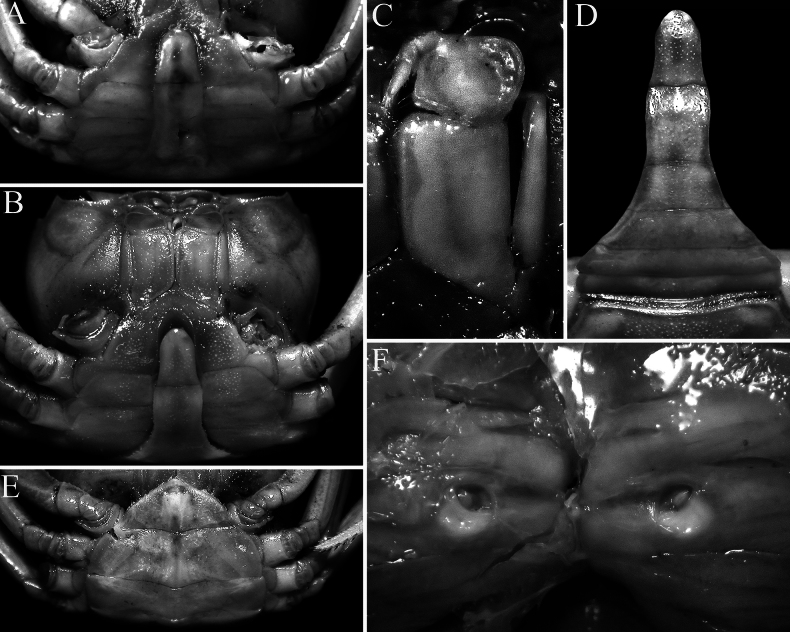
*Stygothelphusa nobilii* (Colosi, 1920). **A**. Lectotype ♂ (15.0 × 13.0 mm) (MRSN Cr 1308a); **B–D**. Paralectotype ♂ (17.0 × 14.7 mm) (MRSN Cr 1308b); **E, F**. Paralectotype ♀ (21.0 × 18.0 mm) (MRSN Cr 1308b). All specimens from Saribau, Sarawak. **A, B**. Ventral view of cephalothorax; **C**. Left third maxilliped; **D**. Male pleon; **E**. Female pleon; **F**. Sternopleonal cavity and vulvae.

**Figure 4.**
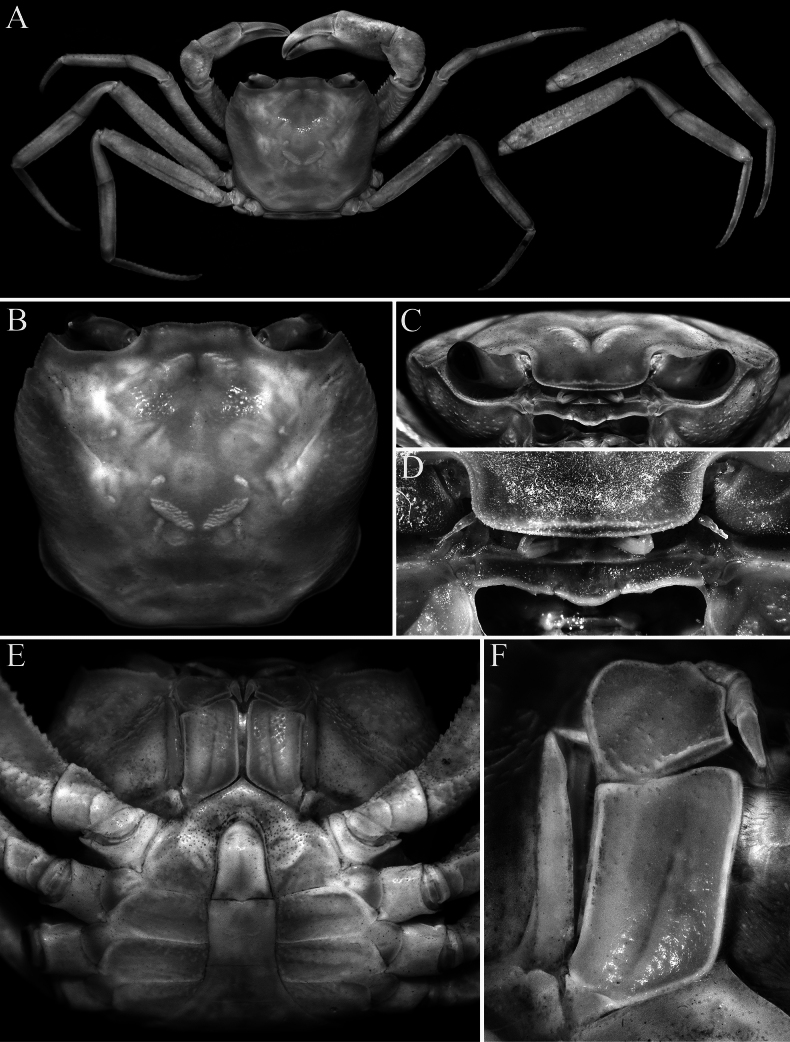
*Stygothelphusa nobilii* (Colosi, 1920), ♂ (17.6 × 15.5 mm) (ZRC 2021.0661). **A**. Overall dorsal habitus; **B**. Dorsal view of carapace; **C**. Frontal view of cephalothorax; **D**. Frontal margin, antennules, antennae and epistome; **E**. Ventral view of cephalothorax; **F**. Right third maxilliped.

**Figure 5.**
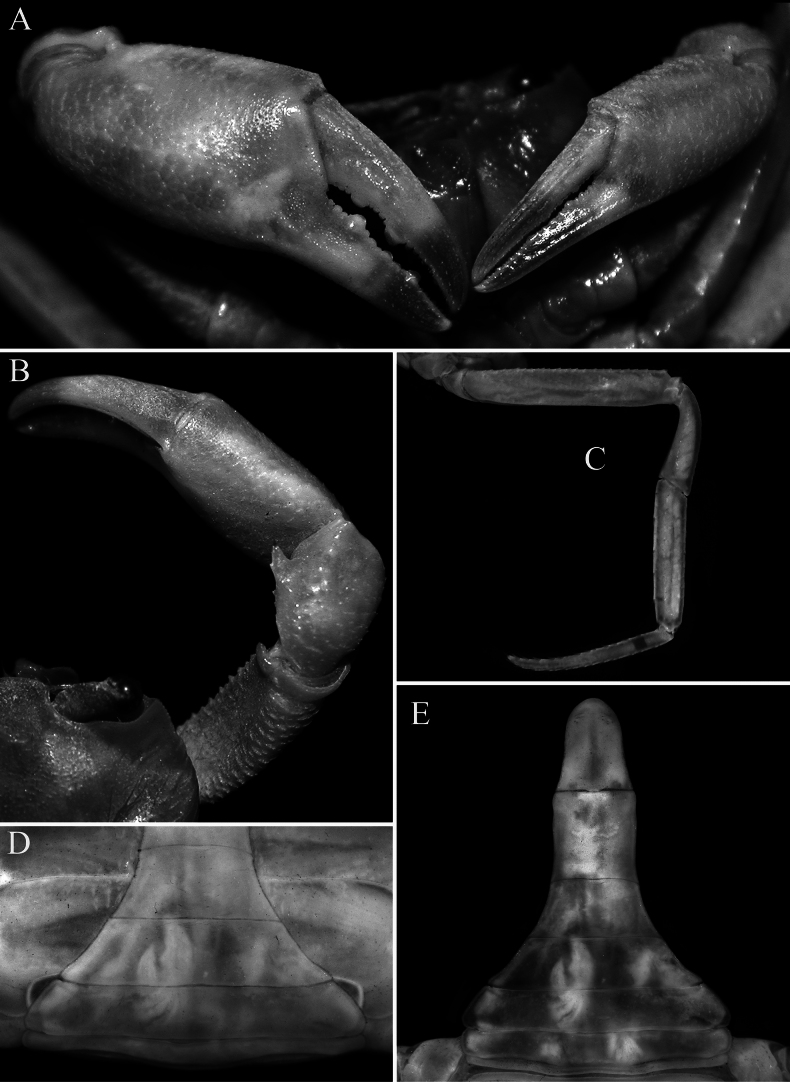
*Stygothelphusa nobilii* (Colosi, 1920), ♂ (17.6 × 15.5 mm) (ZRC 2021.0661). **A**. outer view of chelae; **B**. Dorsal view of right cheliped; **C**. Right P5; **D**. Posterior thoracic sternum and pleon; **E**. Pleon.

**Figure 6.**
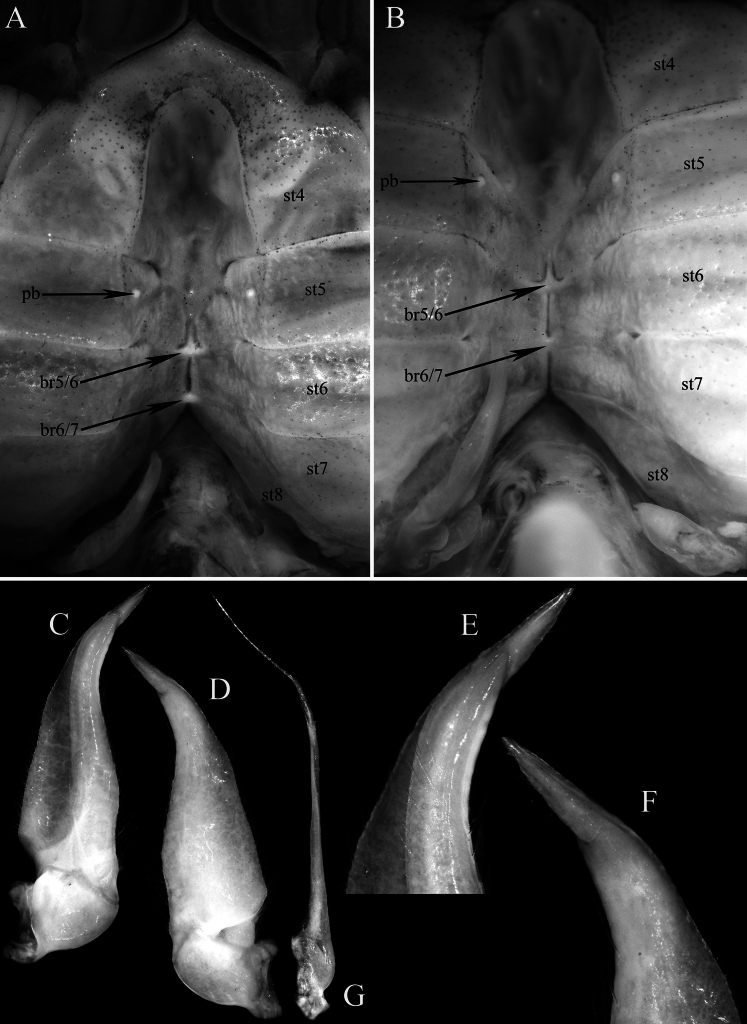
*Stygothelphusa nobilii* (Colosi, 1920), ♂ (17.6 × 15.5 mm) (ZRC 2021.0661). **A, B**. Sternopleonal cavity from different angles; **C**. Left G1 (ventral view); **D**. Left G1 (dorsal view); **E**. Distal part of left G1 (ventral view); **F**. Distal part of left G1 (dorsal view); **G**. Left G2 (dorsal view). Abbreviations: br5/6 = transverse sternal bridge between sternites 5 and 6; br6/7 = transverse sternal bridge between sternites 6 and 7; pb = tubercle of press-button of male pleonal locking mechanism; st4–st8 = thoracic sternites 4–8, respectively.

**Figure 7.**
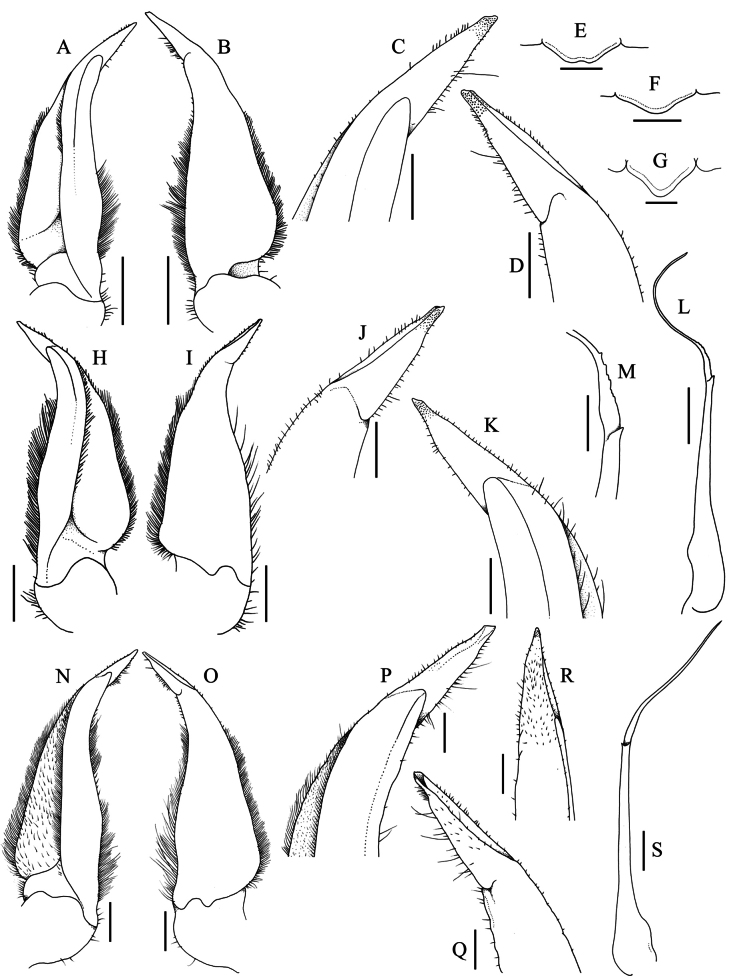
*Stygothelphusa nobilii* (Colosi, 1920). **A–E**. Lectotype ♂ (15.0 × 13.0 mm) (MRSN Cr 1308a); **H–M, F**. Paralectotype ♂ (17.0 × 14.7 mm) (MRSN Cr 1308b); **G**. paralectotype ♀ (21.0 × 18.0 mm) (MRSN Cr 1308b); **N–S**. ♂ (17.6 × 15.5 mm) (ZRC 2021.0661). **A, N**. Left G1 (ventral view); **B, O**. Left G1 (dorsal view); **C, P**. Distal part of left G1 (ventral view); **D, Q**. Distal part of left G1 (dorsal view); **E–G**. Median lobe of posterior epistomal margin; **H**. Right G1 (ventral view); **I**. Right G1 (dorsal view); **J**. Distal part of right G1 (ventral view); **K**. Distal part of right G1 (dorsal view); **L**. Right G2; **M**. Median part of right G2; **R**. Distal part of left G1 (ventro-mesial view); **S**. Left G2 (ventral view). Scale bars: 0.5 mm (**A**, **B**, **E–G**, **H**, **I**, **L**, **N**, **O**, **S**); 0.2 mm (**C**, **D**, **J**, **K**, **M**, **P–R**).

**Figure 8.**
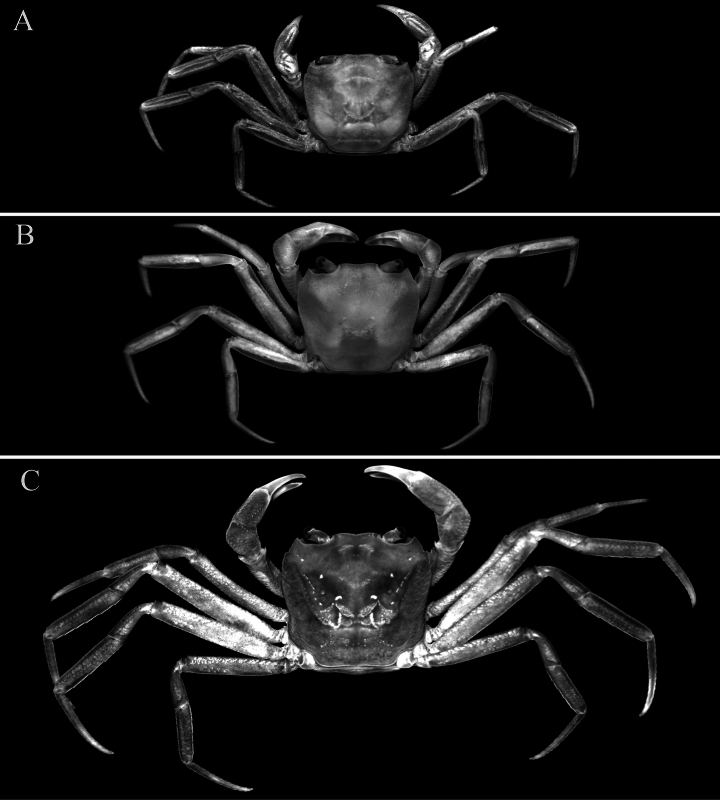
*Stygothelphusa nobilii* (Colosi, 1920), overall dorsal habitus. **A**. ♀ (7.8 × 7.0 mm) (ZRC 2021.0660); **B**. ♀ (12.3 × 11.0 mm) (ZRC 2021.0661); **C**. ♀ (16.2 × 14.5 mm) (ZRC 2017.1276).

**Figure 9.**
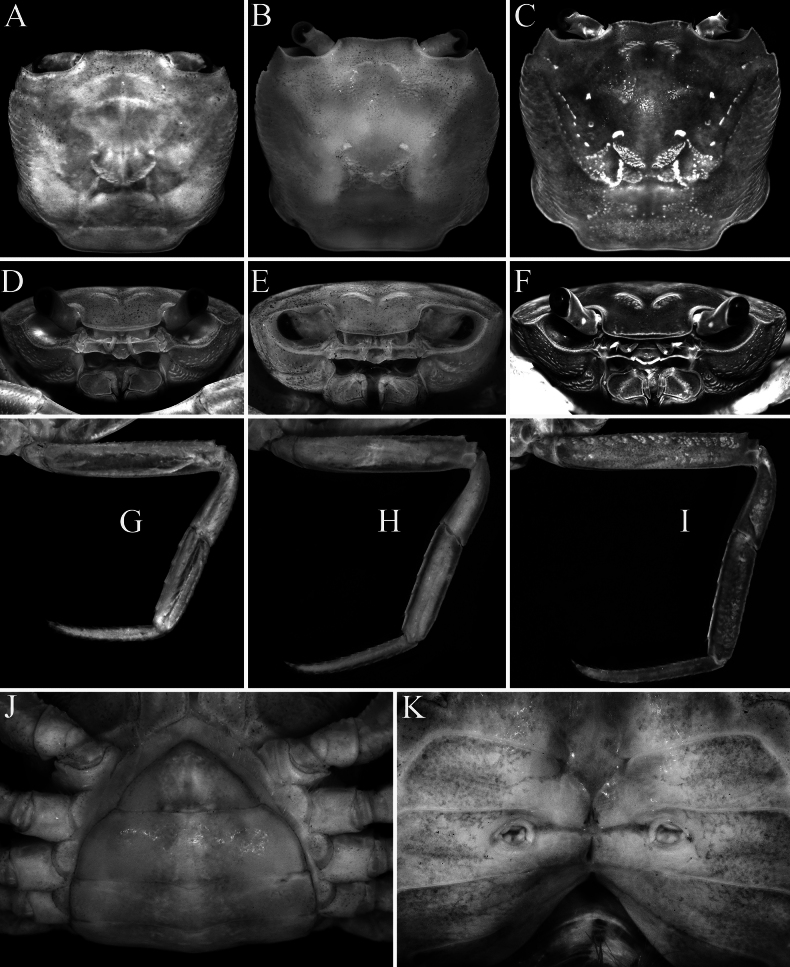
*Stygothelphusa nobilii* (Colosi, 1920). **A, D, G**. ♀ (7.8 × 7.0 mm) (ZRC 2021.0660); **B, E, H**. ♀ (12.3 × 11.0 mm) (ZRC 2021.0661); **C, F, I–K**. ♀ (16.2 × 14.5 mm) (ZRC 2017.1276). **A–C**. Dorsal view of carapace; **D–F**. Frontal view of cephalothorax; **G–I**. Right P5; **J**. Pleon; **K**. Sternopleonal cavity and vulvae.

Carapace quadrate, with lateral margins gently convex, broader than long, width to length ratio 1.10–1.20; dorsal surfaces gently rugose, especially along margins, other parts mostly smooth (Figs 1, 2A, B, 4A, B, 8, 9A–C); striae on anterolateral regions distinct (Figs 1, 2A, B, 4A, B, 8, 9A–C); branchial regions gently inflated dorsally and laterally, lateral margins appearing gently convex from dorsal view, gently convex from frontal view (Figs 1, 2A–E, 4B, C, 9A–F); epibranchial tooth low but visible, separated from external orbital angle by shallow cleft (Figs 1, 2A, B, 4B, 9A–C). Ambulatory legs long, fourth ambulatory merus 0.69–0.81 ×carapace length (Figs 1A, 1B, 2G, 4A, 5C, 8, 9G–I). Male sternopleonal cavity reaching to level of junction between thoracic sternites 2 and 3, on imaginary line connecting anterior margins of coxae of chelipeds (Figs 3A, 3B, 4E). Male pleon T-shaped (Figs 3D, 5D, 5E). G1 curved outwards; terminal article cylindrical, relatively short, ca. 0.23–0.26 ×subterminal article, tip truncate (Figs 6C–E, 7A–D, 7H–K, 7N–R). G2 distal article flagelliform, just shorter than basal article (Figs 6G, 7L, 7S).

##### Colour.

**Figure 10.**
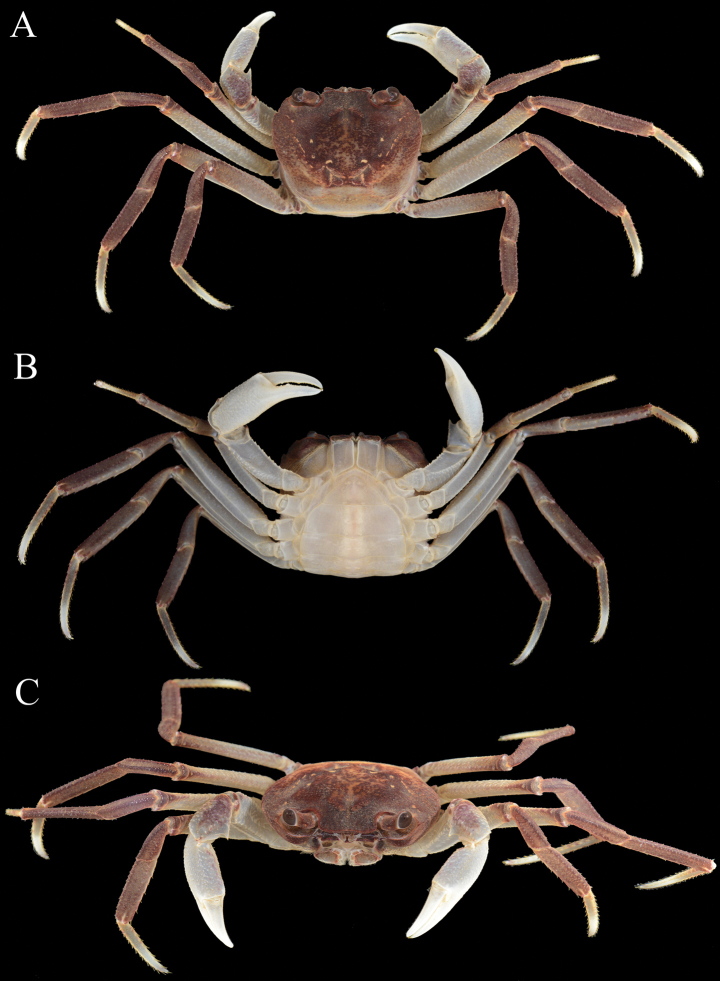
*Stygothelphusa nobilii* (Colosi, 1920), ♀ (16.2 × 14.5 mm) (ZRC 2017.1276). **A**. Overall dorsal habitus; **B**. Ventral view; **C**. Frontal view of cephalothorax.

**Figure 11.**
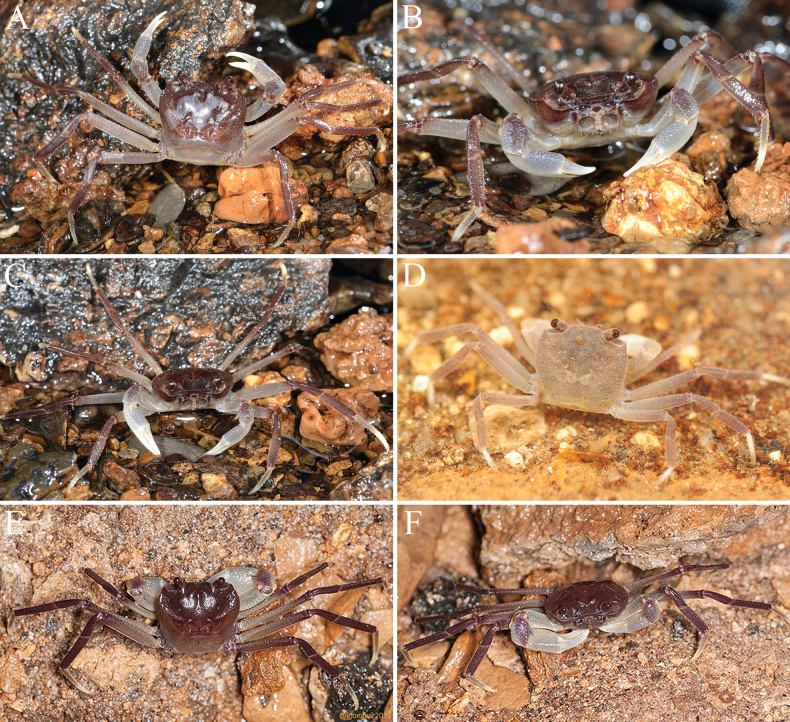
*Stygothelphusa nobilii* (Colosi, 1920). **A–C**. ♀ (16.2 × 14.5 mm) (ZRC 2017.1276); **D**. ♀ (7.8 × 7.0 mm) (ZRC 2021.0660); **E, F**. ♂ (17.6 × 15.5 mm) (ZRC 2021.0661).

Adults with carapace uniformly brown, sometimes with posterior one-third paler; sub-branchial, subhepatic and epistome brown with other surfaces dirty white; chelipeds white with carpus mottled brown; ambulatory legs generally brown with merus paler, sometimes being beige with distal half of dactylus white or yellowish-white (Figs 10, 11A–C, E, F); ventral surfaces dirty white. Young specimens more uniformly yellowish-brown with chelae and ambulatory dactyli white (Fig. 11D).

##### Remarks.

Nobili (1903: 15) recorded Potamon (Geothelphusa) kenepai from Sarawak on the basis of two males and a female specimen collected by Robert Shelford from “Mt Saribau” at an elevation of 762 m (2,500 feet). Colosi (1920) re-examined this material, noted that it was certainly not *P. kenepai*, and referred the specimens to a new species, Parathelphusa (Liothelphusa) nobilii. Colosi (1920: 26) further commented that although the material resembled *Pot.* (*Geot*.) kenepai, it differed distinctly in several important characters, including the greater length of the pereopods, the size and shape of the chelipeds, and the angle formed by the inner orbital margins with the frontal margin. He also suggested affinities with Potamon (Thelphusa) bidiense [= Potamon (Geothelphusa) bidiens Rathbun] from Borneo and *Pot.* (*Geot*.) *araneus* Rathbun from French Indochina but noted that a proper comparison was not possible due to missing diagnostic characters.

Bott (1970: 59) synonymised Parathelphusa (Liothelphusa) nobilii with *Thelphusula hendersoniana*, originally described under Potamon (Geothelphusa), without explanation. Under “material examined”, he listed two males and one female of *Parathelphusa nobilii*, recorded as from “Sarawak, Mt. Sarinau”, and designated one male as the lectotype. Because he did not specify which male specimen was selected, this designation is invalid. It is also unclear whether Bott examined the specimens beyond the gonopods; the ambulatory legs of *T. hendersoniana* are only about half the length of those of *P. nobilii*, making it difficult to reconcile both taxa under a single species if examined side-by-side. It seems unlikely that Bott would have treated them as one if he had specimens of both together. Bott (1970) also did not illustrate the gonopods of *P. nobilii*. The taxonomic confusion is compounded by the fact that the male second gonopod of *T. hendersoniana*, which bears a long, flagellum-like distal article, is entirely different from that of typical species of *Thelphusula* Bott, 1969, which possess a short distal article (cf. Bott 1970: pl. 27 figs 37–40; Tan and Ng 1998: fig. 1G). For these reasons, Ng (1995) transferred Potamon (Geothelphusa) hendersonianum to his new genus *Bakousa* Ng, 1995 (type species *B. sarawakensis* Ng, 1995), together with Potamon (Geothelphusa) kenepai, which Bott had inexplicably placed in *Adeleana* Bott, 1969. In his later revision of *Adeleana*, Ng (2025: 20) reaffirmed this action.

Colosi’s (1920) comparison of *Parathelphusa nobilii* with Potamon (Thelphusa) bidiense was insightful, as both species are indeed closely related. Bott (1970) synonymised *Potamon bidiense* with *T. melanippe* De Man, 1899, but Ng (1989a) demonstrated that *P. bidiense* was distinct and established the new genus *Stygothelphusa* Ng, 1989 for it. Ng (1991) disagreed with Bott’s synonymy of *P. nobilii* with *T. hendersoniana* and provisionally referred *P. nobilii* to *Arachnothelphusa* Ng, 1991, primarily on account of its long ambulatory legs. Ng and Alvarez (2000) subsequently transferred it to *Stygothelphusa* after examining photographs of the types, although no figures were provided. Two additional species of *Stygothelphusa* have since been described from Sarawak, *S. cranbrooki*, and *S. antu*. In describing *S. cranbrooki*, Ng (2013) provided photographs of the three type specimens of *Parathelphusa nobilii* for the first time and affirmed its placement in *Stygothelphusa*, although information on the gonopods was still unavailable.

Our re-examination of the type specimens confirms that the gonopod structures of *P. nobilii* are fully consistent with those of species assigned to *Stygothelphusa*. They are also clearly distinct from congeners by having a relatively shorter terminal article (~0.25 × the subterminal article), straight and with a truncate tip (Figs 6C–F, 7A–D, 7H–K, 7N–R, 13E).

Ng (2013: 93) selected the most complete male (15.0 × 13.0 mm, MRSN Cr 1308a) of *P. nobilii* as the lectotype (Fig. 1A). Unfortunately, both G2s of the specimen are broken and only the left G1 is figured (Fig. 7A–D). The larger paralectotype male (15.0 × 13.0 mm, MRSN Cr 1308b) has the left G1 and G2 detached and missing from the bottle; only the right G1 and G2 were drawn (Fig. 7H–M). We believe that this was the specimen examined by Bott (1970), as it was his practice to detach and retain gonopods of examined species, and these may not have been returned to Turin. The female paralectotype (20.1 × 17.0 mm, MRSN Cr 1308b) has damage to the right branchial region (Fig. 1C); the published figure in Ng (2013: fig. 2B) was inadvertently inverted laterally during printing.

The present specimens of *S. nobilii* from Gua Chupak match the type series closely in all respects, particularly in the diagnostic G1 morphology, which has a relatively short terminal article with a truncate tip (Fig. 7A–D, H–K, N–R). The G1 of the large recent male (17.6 × 15.5 mm, ZRC 2021.0661) (Fig. 7N–R) agrees very well with that of the lectotype (Fig. 7A–D). The G1 of the paralectotype male differs slightly in having a more slender distal terminal article and a proportionately broader subterminal article (Fig. 7H–K), but these differences fall within expected intraspecific variation.

*Stygothelphusa nobilii* can be distinguished from *S. bidiensis* and other congeners (Fig. 13B–D) by its proportionally shorter ambulatory legs (Fig. 13A), a key diagnostic feature emphasized by Colosi (1920) and Ng (2013). In *S. nobilii*, the fourth ambulatory merus measures 0.69–0.81 × the carapace length, whereas in other species of *Stygothelphusa*, the corresponding merus is 0.86–1.03 × the carapace length. Although larger *S. nobilii* have proportionally slightly longer ambulatory legs (cf. Fig. 9G–I) (as is also the case for *S. bidiensis* and *S. cranbrooki*, cf. Ng 2013), they never reach the proportions observed in related species.

The median lobe of the epistomial margin of *S. nobilii* shows some variation. In the lectotype male, the tip is bilobed (Figs 2C, 7E), is rounded in the paralectotype male (Figs 2D, 2E, 7F) and more acute in the paralectotype female (Fig. 7G). In the recent series of specimens from Gua Chupak, the lobe ranges from weakly to distinctly bilobed (Figs 4C, 4D, 9D–F).

**Figure 12.**
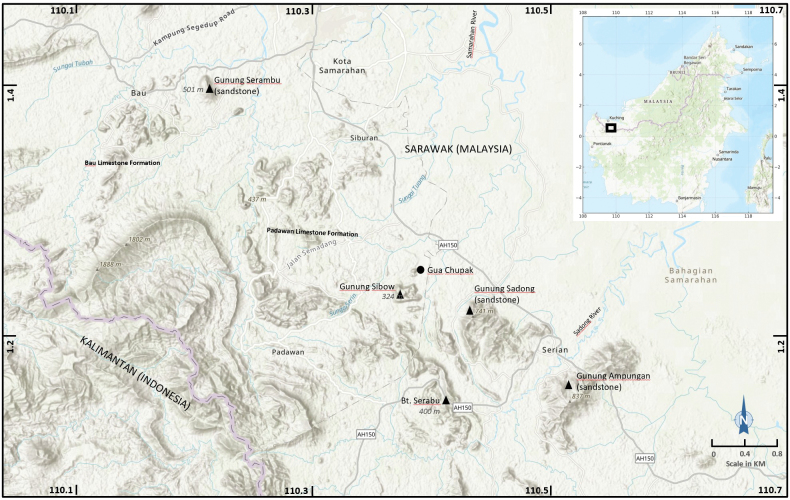
Location of Chupak Cave (= Gua Chupak) in relation to the highest sandstone summit of Gunung Sadong, ca 8 km away in a straight line.

**Figure 13.**
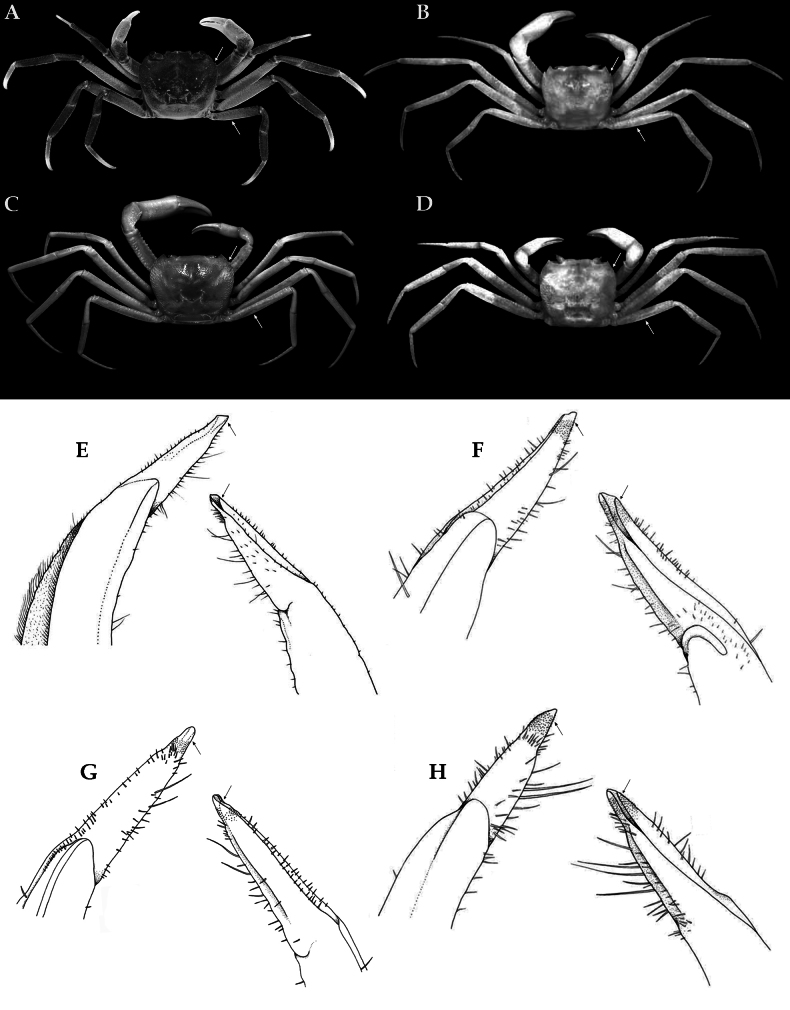
Comparison of external morphological and G1 differences between four species of *Stygothelphusa*. **A, E**. *S. nobilii*, ♂ (17.6 × 15.5 mm) (ZRC 2021.0661); **B, F**. *S. cranbrooki* (after Ng 2013: figs 3A, 6F, G); **C, G**. *S. antu* (after Ng and Grinang 2014: figs 1A, 3C, D); **D, H**. *S. bidiensis* (after Ng 2013: figs 1B, 6B, C). Arrows indicate diagnostic characters discussed in text.

The G1 terminal article of the paralectotype male (17.0 × 14.7 mm, MRSN Cr 1308b) is more conical, with the lateral margins slightly more convergent towards the tip, and the subterminal article is relatively broader as well (Fig. 7H–K). The differences from the lectotype male (15.0 × 13.0 mm, MRSN Cr 1308a) and recent male of *S. nobilii* (17.6 × 15.5 mm, ZRC 2021.0661), however, are slight (cf. Fig. 7A–D, N–R) and within the expected range of intraspecific varaiation. The proximal margin of the G2 distal article of the paralectotype male (MRSN Cr 1308b) has small uneven serrations (Fig. 7M), which are atypical and appear to be anomalous. They are not present on the recent male of *S. nobilii* from Chupak (ZRC 2021.0661) (Fig. 7S) nor on the G2 of congeners.

##### On the type locality, “Mt. Saribau”.

The name of the type locality, “Mount Saribau” (cf. Nobili 1903: 15) – spelled “Sariban” by Colosi [1920: 26] and “Sarinau” by Bott [1970: 59]), presents a problem, as it is not in use today. Colosi’s original labels contain no further information, and there are no labels by Shelford or Nobili in the bottle. No date is associated with the original label, so the exact time of collection is unknown. What is known is that Robert Shelford served as curator of the Sarawak Museum from 1897–1906 and made extensive collections in western Sarawak during this period (Shelford 1916). As Nobili’s (1903) paper was published on 18 July 1903, the specimens were probably collected in 1902 and subsequently sent to Italy.

With regard to the place name, the first edition of the U.S. Board on Geographic Names (Anonymous 1955: 186) list a locality “Saribau: 1°10'N, 111°38'E”, and a later edition (Anonymous 1970: 916) records “Tanjung Saribau 1°10'N, 111°38'E”. This locality lies in central Sarawak, close to Kalimantan boarder, east of Batang Ai National Park. There is also an area called Saribas in central Sarawak (Betong Division), centred on the Saribas River (ca 1°38'N, 111°12'E). Based on what we know about Shelford’s surveys, however, there is no evidence that he explored central Sarawak (Lingga/Sri Aman/Sibu), making a central locality unlikely (see Shelford 1916). Furthermore, there is no “Saribau”, “Sariban” or “Sarinau” listed in the Sarawak Gazetteer (Mohizah et al. 2006).

Review of the literature shows that the name “Mt. Saribau” was explicitly used by Shelford (1902a) in a list of Bornean reptiles, and in an addendum he (Shelford 1902b: 134) noted specimens of two snake species from “Mt. Saribau, Samarahan R.”. Later, Shelford (1905: 209) described a new frog, *Rana sariba*, from Sarawak, stating that his material was from “Mount Saribaw, Samarahan River, Sarawak” (Shelford 1905: 210). This frog is now regarded as a synonym of *Ingerana baluensis* (Boulenger, 1896) (see also Dubois 1987; Inger and Tan 1996). Moulton (1905: 189, 190) also reported two beetle species from “Mt. Saribau”. Stork (1986: 5), in compiling Borneo beetle localities (including those of Moulton 1905), listed “Saribau Mts. Sarawak: 1°17'N, 112°12'E”. These coordinates place the site near the Kalimantan border east of Batang Ai National Park, near Nanga Biru in the Lubok Antu area. It is unclear how Stork (1986) derived these coordinates, but he may have followed the American gazetteers (Anonymous 1955, 1970). Notably, Shelford (1916), in his memoir on his time in Sarawak, did not mention “Mt. Saribau”.

Sheldon et al. (2023: 20), in gazetteer for Sarawak ornithology, recorded “Gunung Serumbu (= Serambu, Sirambu, Serembu, Sirambau, Sirambo, Saribu, Serambo): 1.4199 110.2246 490 m. Museums: MCSNG, NHMUK, SMK, YPM. Collectors: O. Beccari and G. Doria, A.H. and H.H. Everett, Junaidi. References: St. John (1862), Wallace (1869), Sharpe (1877), Everett (1889), Bartlett (1896), Beccari (1904), Moulton (1914). Remarks: Gn. Serambu is the location of Rajah James Brooke’s holiday cottage, called “Peninjau”, meaning look-out.” From the variant names for Gunung Serambu, especially “Sirambu” and “Saribu”, it is plausible that Mt. Saribau may be an old or misspelt name for what is now called Gunung Serambu (ca 1°25'N, 110°14'E). Gunung Serambu, however, is composed largely of porphyry diorite rock of igneous origin and lies on the northern edge of a major karst system to its south. While the neighbouring limestone areas provide suitable habitats for *Stygothelphusa* species, the mountain itself is not limestone, and surveys there have not yielded any obligate karst crabs, although other taxa are present (see Grinang et al. 2016).

In addition, there is also a well-known mining site in the Bau area, the Sarabau Mine (1°24'34"N, 110°10'20"E), situated further west of Gunung Serambu. The limestone hills in this area belong to the Wind and Fairy Cave systems (Wilford 1964), which form the type locality of another species, *S. bidiensis*.

The critical question, therefore, is: where is “Mt. Saribau”? Shelford’s locality data are crucial, as he collected the material used by Nobili (1903) and Colosi (1920). His statements (Shelford 1902b: 134; 1905: 210) that “Mt. Saribau” lies by the Samarahan River are particularly important. The Samarahan River (now Sungei Sabang) rises in the extensive limestone hills around Kampung Chupak and Gunung Sibow (Fig. 12). In contrast, Gunung Serambu lies further north and is associated instead with headwater of the Sarawak River (Fig. 12). For Shelford to place “Mt. Saribau” on the Samarahan River strongly suggest that his collecting locality was in the vicinity of Gunung Sibow, or on that hill itself.

There is an additional problem with Shelford’s data for *S. nobilii*: he recorded the specimens as collected at 2,500 feet (= 762 m), an elevation that appears unrealistically high. Gunung Sibow is just higher than 324 m asl, and the other limestone hills in the Chupak area are consistently lower (Fig. 12). Even Gunung Serambu is only 452 m. The nearest peak approaching this height is Gunung Sadong (Bung Sadong) in the Sadong catchment, further east, with a summit of ca 741 m asl; however, it is composed of sandstone, and our recent surveys revealed no evidence of cave system there. We therefore regard Shelford’s altitude record as erroneous.

It is also uncertain whether Shelford personally collected the *S. nobilii* specimens or whether they were obtained for him by local assistants. Some localities provided by Shelford for other Sarawak crabs are imprecise and likely represent nearby sites rather than exact collecting points (see Grinang and Ng 2015a for *Terrathelphusa kuchingensis* (Nobili, 1901)).

Taking all available evidence altogether, we consider it most likely that “Mt. Saribau” refers to the area now known as Gua Chupak, including Gunung Sibow (Fig. 12). This is an extensive limestone region where local communities have harvested swiftlet nests for centuries and would have been readily accessible to Shelford and/or his collectors. It is precisely from this area that the recent specimens of *S. nobilii* were obtained. Given the ecological requirement of *Stygothelphusa* for limestone habitats, and the close morphological agreement between the recent materials and the type specimens (see earlier remarks), the Chupak–Gunung Sibow area is the most plausible type locality for *S. nobilii*.

## Discussion

*Stygothelphusa nobilii* is known exclusively from cave habitats, like all other congeners; however, unlike typical troglobitic crabs, its eyes and pigmentation are not reduced. The species is therefore best regarded as a recent troglobite, as no populations have yet been found outside cave environment (see Ng 1989a, 2013; Ng and Yussof 1990; Ng and Grinang 2014). Among congeners, *Stygothelphusa nobilii* is distinctive in its comparatively strong pigmentation: adults possess a brown carapace and ambulatory legs (Fig. 11A–C, E, F), whereas juveniles are yellowish white (Fig. 11D). In contrast, the other three species of *Stygothelphusa* remain pale cream or yellowish white throughout life.

Current observations suggest that the population of *S. nobilii* is extremely small, confined to only two highly localized sites within the completely dark zones of Gua Chupak. Individuals are usually encountered during the wet season, implying that seasonal moisture levels influence their surface activity and detectability. The species occupies damp substrates near small intermittent pools or trickling water, frequently taking refuge beneath rocks and within narrow crevices where humidity remains consistently high. The crabs are highly sensitive to light, retreating immediately when illuminated by the beam of a torch. Within this microhabitat, *S. nobilii* co-exists with a pinkish isopod species that is far more abundant. The diet of *S. nobilii* remains unknown, but isopods, detritus, and guano from bats and swiftlets are likely food sources. The extremely restricted distribution and low abundance of the species highlight its ecological specialization and potential susceptibility to microhabitat disturbance. Although the limestone cave systems in the Gua Chupak area lack formal legal conservation protection, they are currently safeguarded de facto under Bidayuh customary laws, which limit quarrying and large-scale development. Nevertheless, the increasing interest in limestone cave-based tourism may pose future threats to the *Stygothelphusa* population if not manage carefully. Given its narrow range, specialized habitat requirements, and limited population size, it is appropriate to regard members of the genus as at least vulnerable to extinction (cf. Cumberlidge et al. 2009).

### Key to species of *Stygothelphusa*

**Table:** 

1	Carapace anterolateral margin with prominent, acutely triangular epibranchial tooth, separated from anterolateral margin by deep V-shaped cleft (Fig. 13B)	***S. cranbrooki* (Gua Sireh, Sadong catchment, Serian Division)**
–	Carapace anterolateral margin unarmed and entire, or with low epibranchial tooth, separated from anterolateral margin by shallow cleft (e.g., Fig. 13A)	**2**
2	Carapace anterolateral margin without obvious epibranchial tooth, appearing entire (Fig. 13D). Ratio of fourth ambulatory merus to carapace length 0.86–0.96 (Fig. 13D)	***S. bidiensis* (Gua Pari-Pari, Bau, Sarawak Kanan catchment, Kuching Division)**
–	Carapace anterolateral margin with low epibranchial tooth but always visible, separated from anterolateral margin by shallow cleft (Fig. 13A)	**3**
3	Carapace more quadrate, carapace width to length ratio 1.10–1.20. Ambulatory legs relatively shorter, ratio of fourth ambulatory merus to carapace length 0.69–0.81 (Fig. 13A)	***S. nobilii* (Gua Chupak, Samarahan catchment, Samarahan Division)**
–	Carapace more transversely rectangular, carapace width to length ratio 1.22–1.23. Ambulatory legs relatively longer, ratio of fourth ambulatory merus to carapace length 1.01–1.03 (Fig. 13C)	***S. antu* (Gua Rembus, Temurang, Sarawak Kiri catchment, Kuching Division)**

### *Parathelphusa nobilii* Ng, 2014 – a junior primary homonym of Parathelphusa (Liothelphusa) nobilii Colosi, 1920

*Parathelphusa nobilii* is a junior primary homonym of Parathelphusa (Liothelphusa) nobilii. Article 57.2 of the Zoological Code (ICZN 1999) states that “Identical species-group names established for different nominal taxa when originally combined with the same generic name (see also Articles 11.9.3.2 and 57.8.1) are primary homonyms [Article 53.3] and the junior name is permanently invalid (but see Article 23.9.5)”. As *Parathelphusa nobilii* is a relatively recent and not widely used, and Parathelphusa (Liothelphusa) nobilii was described after 1899, Article 23.9.5 also cannot be applied to keep the junior name.

We here propose a replacement name, *Parathelphusa daisyae* nom. nov. for *Parathelphusa nobilii*. The species is named after Professor Daisy Wowor, curator of Crustacea at the Bogor Museum and dean of Indonesian freshwater decapod taxonomy. The diagnosis and type series of *Parathelphusa daisyae* nom. nov. from Sambas in Indonesian Kalimantan remain as published by Ng (2014) for this species.

## Supplementary Material

XML Treatment for Stygothelphusa

XML Treatment for Stygothelphusa nobilii

## Additional information

### Use of AI

AI tools were used to retrieve related historical literature from the Biodiversity Heritage Library and Naturalist Institutional Repository. Most old references are not available in our current repository.

### Author ORCIDs

Peter K. L. Ng https://orcid.org/0000-0001-5946-0608

Jongkar Grinang https://orcid.org/0000-0002-3209-5361

### Data availability

All specimens are deposited in museum or institutional collections stated in the paper.
